# Interventions that Facilitate Shared Decision-Making in Cancers with Active Surveillance as Treatment Option: a Systematic Review of Literature

**DOI:** 10.1007/s11912-020-00962-3

**Published:** 2020-07-28

**Authors:** G. E. Collée, B. J. van der Wilk, J. J. B. van Lanschot, J. J. Busschbach, L. Timmermans, S. M. Lagarde, L. W. Kranenburg

**Affiliations:** 1 Department of Psychiatry, Section Medical Psychology and Psychotherapy, Erasmus MC – University Medical Centre, Rotterdam, Netherlands; 2 Department of Surgery, Erasmus MC – University Medical Centre, Dr. Molewaterplein 40 P.O. Box 2040, Suite Na-2119, 3015 GD Rotterdam, Netherlands; 3 Department of Primary and Community Care, Radboud University Medical Centre, Nijmegen, Netherlands

**Keywords:** Active surveillance, Decision aid, Shared decision-making

## Abstract

**Purpose of review:**

Medical decisions concerning active surveillance are complex, especially when evidence on superiority of one of the treatments is lacking. Decision aids have been developed to facilitate shared decision-making on whether to pursue an active surveillance strategy. However, it is unclear how these decision aids are designed and which outcomes are considered relevant. The purpose of this study is to systematically review all decision aids in the field of oncological active surveillance strategies and outcomes used by authors to assess their efficacy.

**Recent findings:**

A search was performed in Embase, Medline, Web of Science, Cochrane, PsycINFO Ovid and Google Scholar until June 2019. Eligible studies concerned interventions aiming to facilitate shared decision-making for patients confronted with several treatment alternatives, with active surveillance being one of the treatment alternatives. Twenty-three eligible articles were included. Twenty-one articles included patients with prostate cancer, one with thyroid cancer and one with ovarian cancer. Interventions mostly consisted of an interactive web-based decision aid format. After categorization of outcomes, seven main groups were identified: knowledge, involvement in decision-making, decisional conflict, treatment preference, decision regret, anxiety and health-related outcomes.

**Summary:**

Although active surveillance has been implemented for several malignancies, interventions that facilitate shared decision-making between active surveillance and other equally effective treatment alternatives are scarce. Future research should focus on developing interventions for malignancies like rectal cancer and oesophageal cancer as well. The efficacy of interventions is mostly assessed using short-term outcomes.

**Electronic supplementary material:**

The online version of this article (10.1007/s11912-020-00962-3) contains supplementary material, which is available to authorized users.

## Introduction

Treatment modalities for cancer include a combination of radiotherapy, chemotherapy and surgery. In addition, active surveillance has been introduced as an alternative treatment option in prostate, colorectal, thyroid and head and neck cancer [1–5, 6•, 7]. In other malignancies such as oesophageal cancer, active surveillance is under investigation as a viable treatment option [8•, 9]. Active surveillance involves frequently performed response evaluations after neoadjuvant therapy using diagnostics (e.g. imaging scans and endoscopic biopsies) to detect remnants of residual disease. Additional treatment is only indicated in those patients with residual disease or progression of disease. Active surveillance strategies have potential advantages, such as the possibility to avoid or delay the need for invasive treatments associated with morbidity and even mortality. However, pitfalls in an active surveillance strategy include the development of an unresectable recurrence, possibly resulting in deterioration of overall survival. Furthermore, distant dissemination rates could theoretically increase due to longer presence of residual tumour in the primary organ, possibly resulting in shedding of tumour cells and development of metastases [10]. In addition, several studies reported that active surveillance induces a certain degree of uncertainty and anxiety for patients, because they might feel like they are living with ‘untreated’ cancer [11–13•]. Finally, the repeated diagnostic measures may also cause a physical burden (e.g. endoscopy) and periodical peaks of anxiety, with possible negative effects on quality of life [14].

Medical decisions concerning active surveillance are often complex, especially because there are multiple treatment options without a clear indication for the best oncological outcome at a group level, let alone at an individual level. The choice of treatment therefore depends on the preferences and values of individual patients as well as their treating physicians. It is preferable that physicians and patients participate in shared decision-making to ensure that the decision made is consistent with the patient’s preferences [15]. Shared decision-making involves informing the patient that a decision is to be made, explaining the potential advantages and disadvantages of each relevant option, discussion of patient’s preferences and finally making the decision together [16]. In order to help patients and physicians making informed decisions together, various interventions have been developed. However, it is unclear how to measure whether these interventions indeed facilitate shared decision-making [17, 18].

In this systematic review, we aim to summarize the design of an intervention and the outcomes that are considered relevant to measure the effectiveness of an intervention used to facilitate shared decision-making in cancer patients for whom active surveillance is a treatment alternative.

## Methods

### Protocol and Registration

The protocol for this study was specified in advance and registered on Prospero (CRD42020139240). The study was performed according to the PRISMA guidelines for systematic reviews [19].

### Eligibility Criteria

Studies were considered eligible if (1) patients were included with malignant disease; (2) on the patients, a choice was imposed between several treatment options, with active surveillance being one of the alternatives; (3) an intervention was used to facilitate shared decision-making; and (4) the outcomes used to measure the effectiveness of the intervention were reported. Interventions were defined as all methods or approaches designed to facilitate involvement in the decision-making process for medical treatment. No restrictions were placed on outcome measures. There was no restriction on publication date. Letters to the editor, editorials, conference abstracts, systematic reviews, narrative reviews and studies written in other languages than English were excluded from further analysis. Also, studies including only patients with palliative options were excluded from further analysis.

### Information Sources and Search

The search strategy was developed in collaboration with an experienced research librarian with an expertise in systematic review searching. The search was applied to Embase and adapted to Medline Ovid, Web of Science, Cochrane Central, PsychINFO Ovid and Google Scholar until June 13, 2019. In addition to these electronic database searches, included papers were checked for relevant references. Search terms included ‘watchful waiting’ or ‘active surveillance’ combined with ‘shared decision’ or ‘decision making’ or ‘patient preference’ or ‘decision aid/tool’ and ‘cancer (treatment)’. The full search strategy is reported in Supplementary Table 1.

Endnote X9 (Thomas Reuters, New York, NY) was used for the reference management of the literature search results. After deduplication, two authors (GC and BvdW) independently screened titles and abstracts of the articles from the search results and selected studies based on the predefined inclusion and exclusion criteria. Inconsistencies were resolved by discussion between the two authors. If no consensus was reached, a third author (LK) resolved any disagreement. The full-text articles were then screened, and motivations for exclusion were recorded. Finally, references of eligible studies were screened for relevance, and references of previously published reviews on this topic were screened for cross-referencing.

### Data Extraction

A data extraction form was developed in order to identify key information and recurring themes within studies. The data extraction form was pilot-tested and refined accordingly. One author (GC) extracted data from included studies, and a second author (BvdW) checked the extracted data. Again, disagreements were resolved by discussion, and if no agreement was reached, a third author made a final decision (LK). Information was extracted from the included studies on (1) characteristics of included participants and studies, including number of patients and type of malignancy as well as the design of the study; (2) type of intervention used; (3) outcomes as measured by authors; (4) instruments used for the assessment of the effectiveness of intervention; and (5) reported results for every outcome. In the present study, the Critical Appraisal Skills Programme (CASP) was used for the assessment of quality of included qualitative studies [20]. For included randomized controlled trials, the risk of bias was assessed using the Cochrane Collaboration’s tool for RCTs, and the ROBINS-I tool was used for assessing risk of bias in non-randomized studies [21, 22].

## Results

### Study Selection

**Fig. 1:**
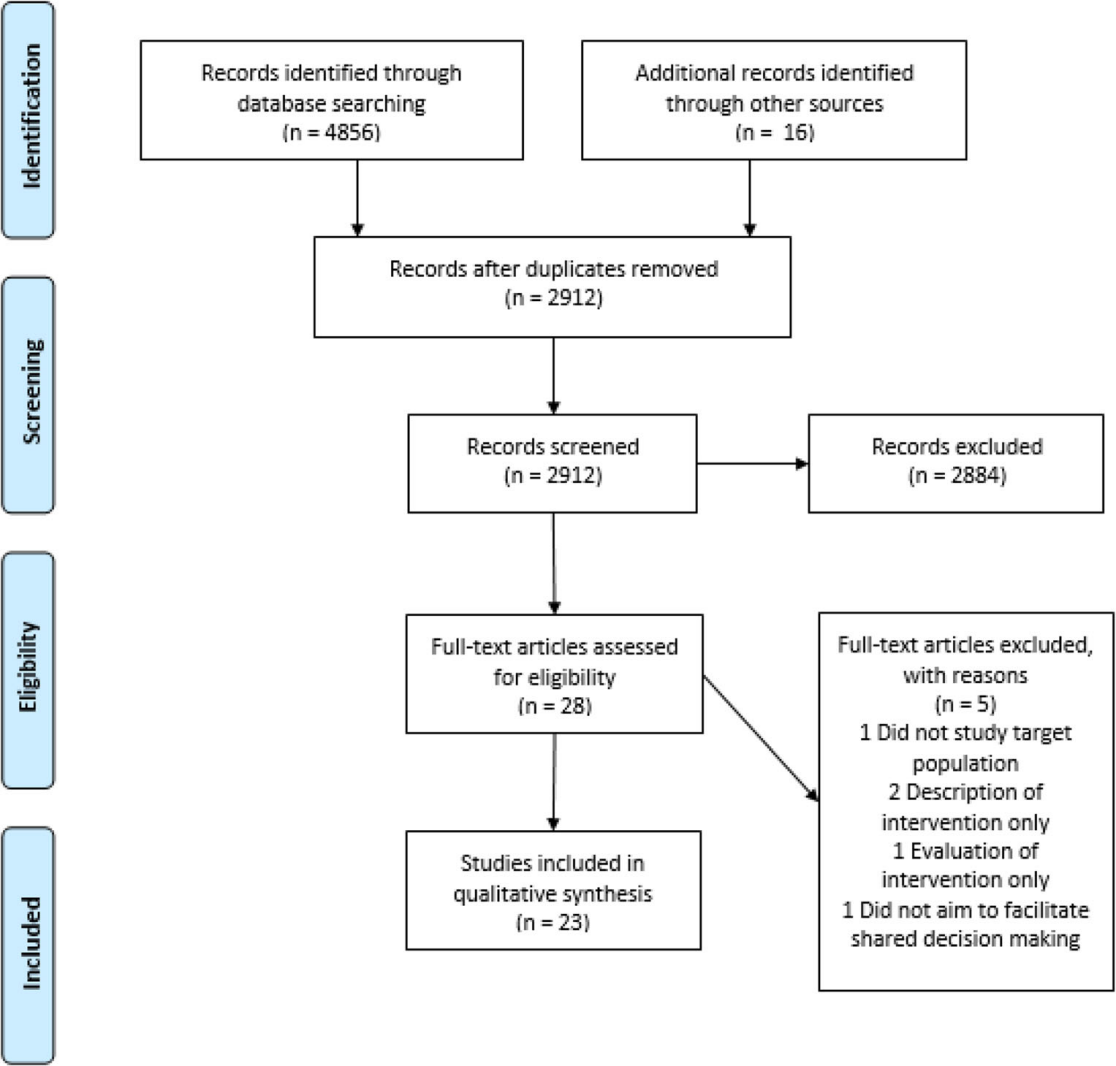
Flow diagram of literature search and study selection

A total of 23 articles, describing 22 unique interventions, were included in this systematic review. From six databases, 4856 articles were identified, and 16 articles were identified through cross-referencing. After adjusting for duplicates, 2912 articles were eligible for title and abstract screening. Of these, 2884 were excluded through title and abstract screening, not meeting the inclusion criteria. After 28 full-text analyses, five additional studies were excluded, ultimately leaving 23 relevant articles. A detailed flowchart for exclusion at each stage and reasons for exclusion after full-text analyses is reported in Fig. 1. Two articles were based on the same trial, but since they measured different outcomes, both studies were included [23, 24]. The results of the risk of bias assessments of all studies are summarized in supplementary Fig. 1a–c. Results and outcomes of the included articles are summarized in Tables 1 and 2.

**Table 1:** Overview of characteristics from 12 randomized controlled trials that were included

First author	Type of cancer	Participants (*N*)	Intervention	Control	Outcome measures
Auvinen, 2004 [25]	Prostate	210	Enhanced participation: emphasis on patient role in decision-making, structured information on treatment options and discussion with physician	Usual care + discussion with physician	Choice of treatment
Feldman-Stewart, 2006 [26]	Prostate	180	Newly developed information booklet	Standard information booklet	Evaluation of DAs, satisfaction with preparation, anxiety, adjustment, decisional conflict
Hack, 2007 [27]	Prostate	425	Audiotape of consult	Usual care	Role in decision-making, communication satisfaction with oncologist, audiotape use and satisfaction, perceived degree of information provision, mood state, cancer-related quality of life
Diefenbach, 2012 [28]	Prostate	72	Internet/CD-ROM-based interactive virtual health centre (with or without tailoring)	Usual care	Evaluation of educational material, decisional variables, treatment preferences
Feldman-Stewart, 2012 [29]	Prostate	156	Decision aid on computer with well-structured information and values clarification exercises	Decision aid with only well-structured information	Decisional conflict, preparation for decision-making, decision regret
Bosco, 2012 [24]	Prostate	448	Computerized decision support system	Standard education + links to websites	Concordance of treatment choice with self-reported influential side effects
Berry, 2013 [23]	Prostate	494	Computerized decision support system	Standard education + links to websites	Decisional conflict, time-to-treatment, treatment choice, program acceptability/usefulness
Hacking, 2013 [30]	Prostate	113	Decision navigation: preparing of personal consultation plan	Usual care	Decisional self-efficacy, decisional conflict, decision regret, mental adjustment to cancer, anxiety and depression, navigation service feedback, final treatment choice
Chabrera, 2015 [31]	Prostate	147	Booklet with information, preparation material for consultation and values clarification exercises	Usual care	Knowledge about prostate cancer, decisional conflict, satisfaction with decision, coping
Song, 2017 [32]	Prostate	156	Video, booklet, tear-out sheet for personal concerns, phone calls to formulate questions	Usual care + handout on staying healthy during treatment	Provision of information, asking questions
Cuypers, 2018 [33]	Prostate	336	Online DA counselling	Standard counselling	Decisional conflict, patients’ perceived role during decision-making, perceived preparedness to make the treatment decision, Pca knowledge, satisfaction with timing and format of the information received, additional questions to evaluate DA
Jayadevappa, 2019 [52]	Prostate	743	Web-based tool for preference assessment	Usual care	Satisfaction with care, satisfaction with decision, decision regret, treatment choice

*RCT* randomized controlled trial, *DA* decision aid, *Pca* prostate cancer

**Table 2:** Overview of characteristics from 11 non-randomized controlled trials that were included

Study	Type of cancer	Participants (*N*)	Intervention	Outcome measures
Onel, 1998 [34]	Prostate	111	Video presentation	Knowledge of prostate cancer, subjective participation in treatment decision, final treatment decision, satisfaction with choice, would choose again
Kim, 2001 [35]	Prostate	30	Interactive CD-ROM decision aid	Prostate cancer knowledge, satisfaction with DA, treatment preference, likelihood of following treatment preference, relationship between Pca knowledge and health literacy
McGregor, 2003 [36]	Prostate	10 healthy men, 12 patients	Video presentation	Insight and knowledge after consultation, communicative effectiveness of video DA, effect of diagnosis on memory and perception, mastery over situation
Feldman-Stewart, 2004 [37]	Prostate	60	Decision aid (one-on-one) interview	Attributes important to the decision, cognitive challenges as determined by patients, changes in important attributes over decision process, changes in treatment ratings, cognitive processes associated with stability of preferred treatment options, cognitive processes associated with regret
Holmes-Rovner, 2005 [38]	Prostate	60	Booklet DA, internet DA and audiotape DA	Different media outcomes, clarity and usefulness of DA, knowledge of pathology results, knowledge of treatment options, discussion of treatment options with physician, active role in treatment decision
Isebaert, 2008 [39]	Prostate	50	Decision aid booklet (based on Holmes-Rovner)	Patients’ general evaluation of the decision aid, final treatment choice, impact of decision aid on treatment choice and consultation according to patients, impact of decision aid on treatment choice and consultation according to doctor
Anderson, 2011 [40]	Ovarian	20	Decision aid booklet	Information and involvement preferences, decision aid feedback, understanding of information contained in DA, difficulties and satisfaction with the decision-making process, anxiety levels
Formica, 2017 [41]	Prostate	452	Video presentation	Knowledge of the rationale for active surveillance
Lamers, 2017 [42]	Prostate	181	Web-based DA with information + values clarification exercises	Concordance of treatment preference before and after DA use, concordance of treatment preference after DA and final choice, concordance initial treatment preference patient and urologist, concordance urologist preference with final decision
Myers, 2018 [43]	Prostate	30	Nurse-mediated online software application	Knowledge about Pca and treatment, patient perceptions regarding Pca and treatment, decisional conflict, treatment preference, treatment status
Brito, 2018 [44]	Thyroid	278	Conversation aid	Final treatment choice

*DA* decision aid, *Pca* prostate cancer

### Study and Patient Characteristics

Of 23 articles included in this study, twelve were randomized controlled trials, which all except one included over 100 patients. Non-randomized trials were mainly cohort studies of which four studies included over 100 patients. Twenty-one articles included patients with prostate cancer, one article included only patients with thyroid cancer and one only patients with ovarian cancer.

### Type of Intervention

In the majority of studies, an interactive web-based decision aid (DA) format was used [23, 24, 28–30, 38, 42, 43, 45]. These DAs included written information, videos and/or exercises offering patients the opportunity to consider what they deemed important regarding the treatment choice of their disease. Six studies used an informational booklet, containing information on the disease, different treatment options and the possible side effects of each treatment option [26, 31, 32, 38–40]. In four studies, a video presentation was the main tool of the DA [32, 34, 36, 41]. In one study, participants received an audiotape DA [38]. Two studies assessed the effect of providing an audiotape of the consultation of the patients with their physician [27, 30]. In five studies, the DA primarily involved an additional consultation with an expert [25, 30, 37, 43, 44]. Three studies explicitly mentioned the added value of clarification exercises to the DA [29, 31, 42]. Please note that some studies did not use only one type of intervention, but a combination of, for example, an information booklet and a web-based DA.

### Effectiveness of Decision Aid

An overview of the different outcomes measured by the authors is offered in Tables 1 and 2. A large heterogeneity exists in these outcomes. In order to acquire more insights into the outcome measures, seven groups were constructed by categorizing the outcomes according to most occurring related outcome measures. These groups are knowledge, involvement in decision-making, decisional conflict, treatment preference/choice, decision regret/satisfaction with decision, anxiety/coping/mood and health-related outcomes. Knowledge was measured in 7 studies, involvement in decision-making in 10 studies, decisional conflict in 9 studies, treatment preference/choice in 13 studies, decision regret/satisfaction with decision in 6 studies, anxiety/coping/mood in 5 studies and health-related outcomes in 1 study.

Four questionnaires were used frequently by different authors: the Preparation for Decision Making Scale, the Decisional Conflict Scale, the Decision Regret Scale and the Satisfaction with Decision Scale. Knowledge and evaluation of DA were often measured with questionnaires developed by the authors. The results of each individual study assessing the effectiveness of the intervention used are summarized in Table 3. Only one study measured outcomes specific to active surveillance, and this outcome was ‘knowledge of the rationale for active surveillance’ [41].

**Table 3:** Categorized outcomes used by authors to assess the effectiveness of the intervention used as well as a summary result described by the authors

Study	Knowledge	Involvement in decision-making	Decisional conflict	Treatment preference/choice	Decision regret/satisfaction with decision	Anxiety/coping/mood	Health-related outcomes
Auvinen, 2004 [25]	n.a.	n.a.	n.a.	58% of men in the intervention group chose the standard treatment vs. 86% in the control group (*p* < 0.001)	n.a.	n.a.	n.a.
Feldman-Stewart, 2006 [26]	n.a.	Patients in the intervention group felt better prepared for decision-making compared with the control group (*p* = 0.047)^a^	Patients in the intervention group appear to experience less decisional conflict^b^	n.a.	n.a.	Anxiety appears lower in the intervention group, and adjustment seems higher, but for both, no significant effect was found	n.a.
Hack, 2007 [27]	n.a.	n.a.	n.a.	n.a.	n.a.	No significant difference in mood state was found between the two groups	Audiotape benefit was not significantly related to patient satisfaction with cancer-related quality of life at 12 weeks post-consultation
Diefenbach, 2012 [28]	n.a.	Patients in the intervention group felt more confident about decision-making	Patients in the intervention group scores lower on decisional conflict^b^	No significant impact of intervention on treatment preferences was found	n.a.	n.a.	n.a.
Feldman-Stewart, 2012 [29]	n.a.	Patients in the intervention group felt better prepared for decision-making at follow-up^a^	Decisional conflict decreased in both groups^b^	n.a.	At > 1-year follow-up, the mean regret of the intervention group was lower (*p* = 0.047)^c^	n.a.	n.a.
Bosco, 2012 [24]	n.a	n.a.	n.a.	45% of men in the intervention group chose treatment in concordance with self-reported influential side effects vs. 50% in the control group	n.a.	n.a.	n.a.
Berry, 2013 [23]	n.a.	n.a.	n.a.	Men in the intervention group chose brachytherapy more often (*p* = 0.01)	n.a.	n.a.	n.a.
Hacking, 2013 [30]	n.a.	Decisional self-efficacy increased in both groups but was higher in the intervention group (*p* = 0.011)	Scores on decisional conflict were lower in the intervention group (*p* = 0.047)^b^	Control group: surgery [22], external beam radiotherapy [17], hormone therapy [2], brachytherapy [2], active monitoring [14] Intervention group: surgery [17], external beam radiotherapy [11], hormone therapy [5], brachytherapy [5], active monitoring [9]	Lower in intervention group at 6-month follow-up (*p* = 0.036)	No significant difference between groups was found for mental adjustment to cancer, anxiety or depression	n.a.
Chabrera, 2015 [31]	Men in the intervention group scored significantly higher on knowledge after DA use compared with the control group (*p* < 0.001)	n.a.	Patients in the intervention group had lower decisional conflict scores (*p* < 0.001)	n.a.	Higher satisfaction with decision scores in intervention group (*p* < 0.001)^4^	Patients in the intervention group made more extensive use of coping mechanisms (*p* < 0.001)	n.a.
Song, 2017 [32]	n.a.	Higher percentages of patients and family members in the intervention group provided information and asked questions during the consult	n.a.	n.a.	n.a.	n.a.	n.a.
Cuypers, 2018 [33]	n.a.	Patients in the intervention group felt less prepared to make the treatment decision^a^	No significant difference between groups^b^	n.a.	n.a.	n.a.	n.a.
Jayadevappa, 2019 [52]	n.a.	n.a.	n.a.	66% of men in the intervention group chose active surveillance vs. 54% in the control group (*p* < 0.001)	Regret declined in both groups, after 24 months intervention group showed less regret (*p* < 0.05); satisfaction improved in both groups, improvement was greater in the intervention group (*p* < 0.05)^d^	n.a.	n.a.
Onel, 1998 [34]	Increase in self-reported knowledge	75 to 84% of patients felt they participated ‘a lot’ in the treatment decision	n.a.	Surgery [39], radiotherapy [40], hormonal therapy [8•], watchful waiting [22]	93% of patients were satisfied with their treatment decision, 100% of patients who chose hormonal treatment were satisfied, whereas 84% of patients who chose surgery were satisfied with their choice	n.a.	n.a.
Kim, 2001 [35]	Mean score of 74%, correlation between knowledge scores and health literacy	n.a.	n.a.	Treatment preferences: hormonal therapy (20%), radiation (13.3%), radical prostatectomy (10%) and combined hormonal and radiation therapy (13.5%). 66.7% received treatments different from those preferences	n.a.	n.a.	n.a.
McGregor, 2003 [36]	Patients reported increased understanding of their disease and its management	Patients felt empowered to take an active role in the decision-making process	n.a.	n.a.	n.a.	n.a.	n.a.
Feldman-Stewart, 2004 [37]	n.a.	n.a.	92% strongly agreed that they were clear about the importance of benefits, 88% strongly agreed that they were clear about the importance of risks and side effects and 47% strongly agreed that it was hard for them to decide whether the benefits or the risks were important to them	76% of men chose the treatment preference that they had at the end of the intervention	Lack of regret after the decision was positively associated with increasing differentiation between treatment options over time	n.a.	n.a.
Holmes-Rovner, 2005 [38]	Intervention group shows some increase in knowledge, especially on watchful waiting and on side effects	Increase in discussion of surgery with physician (*p* = 0.02); 72% of men reported that they were more likely to take an active role in their treatment decision	n.a.	n.a.	n.a.	n.a.	n.a.
Isebaert, 2008 [39]	n.a.	Intervention resulted in more active involvement in decision-making, according to both patient and doctor	n.a.	Radical prostatectomy [19], external beam radiation [14], brachytherapy [10], watchful waiting [6•], 1 remained inconclusive	n.a.	n.a.	n.a.
Anderson, 2011 [40]	n.a.	n.a.	The average decisional conflict score was lower than in comparable samples^b^	n.a.	n.a.	Anxiety scores were high but similar to one comparable study	n.a.
Formica, 2017 [41]	Patients who watched DA had more knowledge of the rationale for active surveillance	n.a.	n.a.	n.a.	n.a.	n.a.	n.a.
Lamers, 2017 [42]	n.a.	n.a.	n.a.	Final treatment choice was in excellent agreement with treatment preference after DA and in good agreement with urologist preference	n.a.	n.a.	n.a.
Myers, 2018 [43]	Increase in knowledge after DA (*p* < 0.001)	n.a.	Decisional conflict scores decreased (*p* < 0.001)^b^	Active surveillance (83%), active treatment (17%)	n.a.	n.a.	n.a.
Brito, 2018 [44]	n.a.	n.a.	n.a.	Patients in intervention group were more likely to choose active surveillance (89% vs. 77% in control group)	n.a.	n.a.	n.a.

^a^Preparation for Decision Making Scale, ^b^Decisional Conflict Scale, ^c^Decision Regret Scale, ^d^Satisfaction with Decision Scale, *DA* decision aid, *n.a.* not applicable

Out of the 23 studies, eleven added the patients’ evaluation of their DA as an outcome measure [23, 26–28, 30, 33, 35, 36, 38–40]. In these studies, patients were asked for their feedback concerning acceptability, feasibility, clarity, usefulness, satisfaction with timing and format of the information and satisfaction with DA in general or communicative effectiveness.

## Discussion

This systematic review presents an overview of interventions aimed at facilitating shared decision-making in cancer patients who are confronted with a treatment choice in which active surveillance is a treatment alternative and the outcomes are considered relevant in this respect. Surprisingly, even though active surveillance is an established treatment alternative also for patients with rectal cancer head and neck cancer and is under investigation for oesophageal cancer, current interventions are mostly limited to patients with prostate cancer. The present study is the first systematic review that provides an overview of outcomes used to test the effectiveness of interventions aimed at facilitating shared decision-making in cancer when active surveillance is a treatment alternative. This resulted in an insight in the spectrum of interventions used, for what purpose and which outcomes have been measured.

Of the 23 included studies, 21 have developed decision aids for patients with prostate cancer. This is remarkable given that active surveillance has also been performed in patients with rectal cancer and head and neck cancer for over 15 years. Furthermore, in several malignancies, an active surveillance strategy has been topic of debate (e.g. oesophageal cancer). A recent systematic review assessed all studies that used decision aids for patients with colorectal cancer [46]. The authors of this study screened 3773 articles and eventually included three articles [47–49]. Of these three articles, two articles used the decision aid to support the decision between chemotherapy or no chemotherapy treatment. One article used the aid to choose between two surgical techniques. No decision aids were developed to support the decision including active surveillance, as is the focus of this systematic review.

The present study reported on 22 unique interventions. It seems that there is no consensus on which type of intervention is most effective. Booklets, videos and web-based DAs are the most commonly used interventions, and more recent studies sometimes included a consultation with a professional to talk about the preferences of the patient. Most interventions rely on the patients’ own motivation to use the decision aid and to improve their understanding of the (dis)advantages of each treatment. As such, patients are expected to return to their physician with a better understanding of their disease after having used the specific DA. Most interventions also encourage the patient to consider their values and preferences. However, it remains unclear to what extent these values and preferences are taken into account in the consultation and final decision-making with the physician.

Finally, there is a large heterogeneity in the outcomes used by authors to assess the effectiveness of the tested interventions. After categorization of the outcomes, treatment choice or preference was most reported to test efficacy of interventions. The reason for this remains unclear, because DAs should not aim to increase the choice for a specific treatment but rather to facilitate shared decision-making by helping patients and their healthcare professionals make a treatment choice best fitted to their unique circumstances [50]. Whether or not the interventions succeeded in this respect is most probably not measured by assessing the treatment choice of the patient. We propose that self-reported involvement in decision-making could be a representative short-term outcome and decisional conflict could be a representative long-term outcome for the effectiveness of DAs. Indeed, self-reported involvement in decision-making was used as outcome in a large number of the articles. Decisional conflict, however, was used as outcome only in a minority of studies. This could be due to the fact that a longer follow-up is needed for this outcome. Even though all studies included in this review had active surveillance as a treatment option, only one study used an outcome measure specific to active surveillance, i.e. knowledge of the rationale for active surveillance [41]. There are usually no outcome measures specific to the other treatment options either; however, active surveillance seems different from the other treatment options. For active surveillance to be successful, it is very important that patients who choose active surveillance understand what it entails for both acceptance and adherence to the active surveillance strategy, as reported in a previous study [51].

The present study is associated with limitations. Firstly, because of the limited variety in malignancies discussed, mostly DAs for prostate cancer were analysed. Consequently, we assessed the outcomes for a selected group of patients, and as such, these results might not be one to one extrapolated to the general population. However, we included only malignancies that also involved active surveillance as treatment alternative, enhancing the generalizability among the malignancies with active surveillance as treatment option. Secondly, due to the large heterogeneity in outcomes used by the authors to assess the effectiveness of the intervention, a categorization of these outcomes was necessary for overview. Inevitably, in this way, interpretation of the results could not be avoided. Lastly, since both patients and physicians are involved in shared decision-making, it would be interesting to gain more insights in the evaluation of the developed interventions from a physician perspective. The current search strategy was not designed to answer this question.

## Conclusion

In conclusion, interventions facilitating the choice between several treatment options with active surveillance as one of the alternatives have been developed mostly for prostate cancer, thus far. The outcomes used to assess the effectiveness of the interventions are highly heterogenic, and it remains unclear how interventions are exactly supposed to facilitate shared decision-making. Future research should focus on developing interventions for malignancies other than prostate cancer, like rectal cancer, head and neck cancer and oesophageal cancer. Furthermore, interventions that facilitate shared decision-making might benefit from more long-term follow-up research, measuring outcomes like decision regret. With active surveillance, patients have to return to the hospital regularly for a few years, and it would be interesting to see how the intervention affects patients after a year or more, especially regarding patient-reported outcomes like anxiety and decision regret.

## Electronic Supplementary Material

ESM 1 (DOCX 12 kb)

ESM 2 (DOCX 116 kb)

ESM 3 (DOCX 126 kb)

ESM 4 (DOCX 12 kb)
