# Supplementary material for: Interventions that Facilitate Shared Decision-Making in Cancers with Active Surveillance as Treatment Option: a Systematic Review of Literature
Source: Curr Oncol Rep. 2020 Jul 28;22(10):101. doi: 10.1007/s11912-020-00962-3 (PMC7387328; doi:10.1007/s11912-020-00962-3)
Supplement: Supplementary file 1 — (DOCX 12 kb) [file 11912_2020_962_MOESM1_ESM.docx]

| **Supplementary Table 1: full search strategy and results until June 13, 2019** | |
| --- | --- |
| **Embase.com** | ('watchful waiting'/de OR 'active surveillance'/de OR (((watch* OR see) NEAR/3 wait*) OR (wait NEAR/3 see) OR (active NEAR/3 surveillan*) OR ('not' NEXT/2 'to treat')):ab,ti) AND ('decision making'/exp OR 'decision support system'/exp OR 'decision tree'/exp OR 'patient preference'/de OR (((decision* OR choice* OR choose OR chose OR wish) NEAR/6 (making OR support* OR system* OR tree* OR shared OR aid OR tool* OR model* OR analy* OR patient* OR informed* OR regret* OR clinical* OR treatment* OR factor* OR affect* OR prefer*)) OR (patients* NEAR/3 (treatment* OR therap*) NEAR/3 (selection* OR prefer*))):ab,ti) AND ('neoplasm'/exp OR 'cancer patient'/de OR 'cancer surgery'/de OR (neoplasm* OR tumo* OR cancer* OR malign* OR carcinom*):ab,ti) AND [English]/lim NOT ([animals]/lim NOT [humans]/lim) |
| **Medline Ovid** | (Watchful Waiting/ OR (((watch* OR see) ADJ3 wait*) OR (wait ADJ3 see) OR (active ADJ3 surveillan*) OR "not to treat").ab,ti.) AND (exp Decision Making/ OR Decision Support Techniques/ OR Decision Trees/ OR Patient Preference/ OR (((decision* OR choice* OR choose OR chose OR wish) ADJ6 (making OR support* OR system* OR tree* OR shared OR aid OR tool* OR model* OR analy* OR patient* OR informed* OR regret* OR clinical* OR treatment* OR factor* OR affect* OR prefer*)) OR (patients* ADJ3 (treatment* OR therap*) ADJ3 (selection* OR prefer*))).ab,ti.) AND (exp Neoplasms/ OR (neoplasm* OR tumo* OR cancer* OR malign* OR carcinom*).ab,ti.) AND english.la. NOT (exp animals/ NOT humans/) |
| **Web of science** | TS=(((((watch* OR see) NEAR/2 wait*) OR (wait NEAR/2 see) OR (active NEAR/2 surveillan*) OR ("not" NEAR/2 "to treat"))) AND ((((decision* OR choice* OR choose OR chose OR wish) NEAR/5 (making OR support* OR system* OR tree* OR shared OR aid OR tool* OR model* OR analy* OR patient* OR informed* OR regret* OR clinical* OR treatment* OR factor* OR affect* OR prefer*)) OR (patients* NEAR/2 (treatment* OR therap*) NEAR/2 (selection* OR prefer*)))) AND ((neoplasm* OR tumo* OR cancer* OR malign* OR carcinom*))) AND LA=(english) |
| **Cochrane CENTRAL** | ((((watch* OR see) NEAR/3 wait*) OR (wait NEAR/3 see) OR (active NEAR/3 surveillan*) OR ('not' NEXT/2 'to treat')):ab,ti) AND ((((decision* OR choice* OR choose OR chose OR wish) NEAR/6 (making OR support* OR system* OR tree* OR shared OR aid OR tool* OR model* OR analy* OR patient* OR informed* OR regret* OR clinical* OR treatment* OR factor* OR affect* OR prefer*)) OR (patients* NEAR/3 (treatment* OR therap*) NEAR/3 (selection* OR prefer*))):ab,ti) AND ((neoplasm* OR tumo* OR cancer* OR malign* OR carcinom*):ab,ti) |
| **PsychINFO Ovid** | ((((watch* OR see) ADJ3 wait*) OR (wait ADJ3 see) OR (active ADJ3 surveillan*) OR "not to treat").ab,ti.) AND (exp Decision Making/ OR Decision Support Systems/ OR (((decision* OR choice* OR choose OR chose OR wish) ADJ6 (making OR support* OR system* OR tree* OR shared OR aid OR tool* OR model* OR analy* OR patient* OR informed* OR regret* OR clinical* OR treatment* OR factor* OR affect* OR prefer*)) OR (patients* ADJ3 (treatment* OR therap*) ADJ3 (selection* OR prefer*))).ab,ti.) AND (exp Neoplasms/ OR (neoplasm* OR tumo* OR cancer* OR malign* OR carcinom*).ab,ti.) AND english.la. NOT (exp animals/ NOT humans/) |
| **Google scholar** | "watchful waiting"\|"wait*see"\|"active surveillance"\|"not to treat" "decision making\|support\|system\|tree\|aid\|tool\|model"\|"patient\|shared decision" neoplasm\|tumor\|cancer\|malignancy\|carcinoma |
