# Supplementary material for: Interventions that Facilitate Shared Decision-Making in Cancers with Active Surveillance as Treatment Option: a Systematic Review of Literature
Source: Curr Oncol Rep. 2020 Jul 28;22(10):101. doi: 10.1007/s11912-020-00962-3 (PMC7387328; doi:10.1007/s11912-020-00962-3)
Supplement: Supplementary file 2 — (DOCX 116 kb) [file 11912_2020_962_MOESM2_ESM.docx]

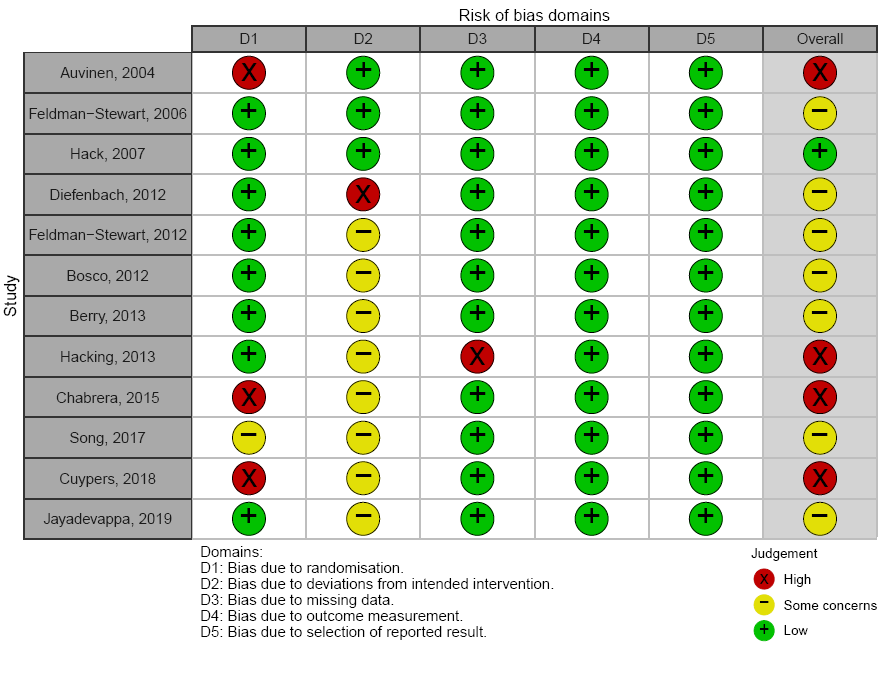


Supplementary Figure 1a – Risk of bias for randomised controlled trials using the Cochrane ROB2-tool
