# Supplementary material for: Interventions that Facilitate Shared Decision-Making in Cancers with Active Surveillance as Treatment Option: a Systematic Review of Literature
Source: Curr Oncol Rep. 2020 Jul 28;22(10):101. doi: 10.1007/s11912-020-00962-3 (PMC7387328; doi:10.1007/s11912-020-00962-3)
Supplement: Supplementary file 3 — (DOCX 126 kb) [file 11912_2020_962_MOESM3_ESM.docx]

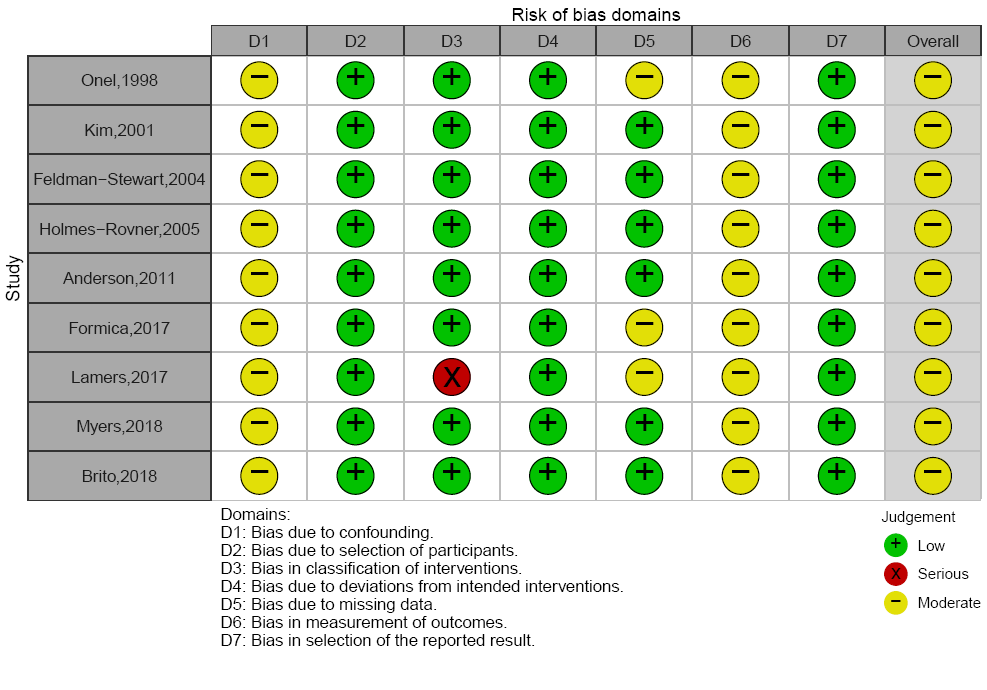


Supplementary Figure 1b – Risk of bias for non-randomised studies using the Cochrane ROBINS-I-tool
