# Supplementary material for: Interventions that Facilitate Shared Decision-Making in Cancers with Active Surveillance as Treatment Option: a Systematic Review of Literature
Source: Curr Oncol Rep. 2020 Jul 28;22(10):101. doi: 10.1007/s11912-020-00962-3 (PMC7387328; doi:10.1007/s11912-020-00962-3)
Supplement: Supplementary file 4 — (DOCX 12 kb) [file 11912_2020_962_MOESM4_ESM.docx]

|  | Section A | | | | | | Section B | | |
| --- | --- | --- | --- | --- | --- | --- | --- | --- | --- |
|  | Q1 | Q2 | Q3 | Q4 | Q5 | Q6 | Q7 | Q8 | Q9 |
| Isebaert, 2009 | Yes | Yes | No | Yes | Yes | No | No | No | Yes |
| McGregor, 2003 | Yes | Yes | Yes | No | No | No | No | No | No |
|  | Q: Question  Section A: Are the results of the study valid? | | | | | | | | |
|  | Section B: What are the results? | | | | | | | | |

Supplementary Figure 1c – Risk of bias for qualitative studies using the CASP checklist
